# Early detection and stratification of lung cancer aided by a cost-effective assay targeting circulating tumor DNA (ctDNA) methylation

**DOI:** 10.1186/s12931-023-02449-8

**Published:** 2023-06-17

**Authors:** Zhoufeng Wang, Kehui Xie, Guonian Zhu, Chengcheng Ma, Cheng Cheng, Yangqian Li, Xue Xiao, Chengpin Li, Jun Tang, Hui Wang, Zhixi Su, Dan Liu, Wengeng Zhang, Yan Huang, Huairong Tang, Rui Liu, Weimin Li

**Affiliations:** 1 Department of Respiratory and Critical Care Medicine, Institute of Respiratory Health, Frontiers Science Center for Disease-related Molecular Network, West China Hospital, Sichuan University, Chengdu, Sichuan China; 2 Singlera Genomics (Shanghai) Ltd, Shanghai, China; 3 Precision Medicine Key Laboratory of Sichuan Province, West China Hospital, Sichuan University, Chengdu, Sichuan China; 4 Health Management Center, West China Hospital, Sichuan University, Chengdu, Sichuan China

**Keywords:** Lung cancer, Early detection, Circulating tumor DNA, Methylation, qPCR assay

## Abstract

**Background:**

Detection of lung cancer at earlier stage can greatly improve patient survival. We aim to develop, validate, and implement a cost-effective ctDNA-methylation-based plasma test to aid lung cancer early detection.

**Methods:**

Case-control studies were designed to select the most relevant markers to lung cancer. Patients with lung cancer or benign lung disease and healthy individuals were recruited from different clinical centers. A multi-locus qPCR assay, LunaCAM, was developed for lung cancer alertness by ctDNA methylation. Two LunaCAM models were built for screening (-S) or diagnostic aid (-D) to favor sensitivity or specificity, respectively. The performance of the models was validated for different intended uses in clinics.

**Results:**

Profiling DNA methylation on 429 plasma samples including 209 lung cancer, 123 benign diseases and 97 healthy participants identified the top markers that detected lung cancer from benign diseases and healthy with an AUC of 0.85 and 0.95, respectively. The most effective methylation markers were verified individually in 40 tissues and 169 plasma samples to develop LunaCAM assay. Two models corresponding to different intended uses were trained with 513 plasma samples, and validated with an independent collection of 172 plasma samples. In validation, LunaCAM-S model achieved an AUC of 0.90 (95% CI: 0.88–0.94) between lung cancer and healthy individuals, whereas LunaCAM-D model stratified lung cancer from benign pulmonary diseases with an AUC of 0.81 (95% CI: 0.78–0.86). When implemented sequentially in the validation set, LunaCAM-S enables to identify 58 patients of lung cancer (90.6% sensitivity), followed by LunaCAM-D to remove 20 patients with no evidence of cancer (83.3% specificity). LunaCAM-D significantly outperformed the blood test of carcinoembryonic antigen (CEA), and the combined model can further improve the predictive power for lung cancer to an overall AUC of 0.86.

**Conclusions:**

We developed two different models by ctDNA methylation assay to sensitively detect early-stage lung cancer or specifically classify lung benign diseases. Implemented at different clinical settings, LunaCAM models has a potential to provide a facile and inexpensive avenue for early screening and diagnostic aids for lung cancer.

**Supplementary Information:**

The online version contains supplementary material available at 10.1186/s12931-023-02449-8.

## Background

Lung cancer remains to be the leading cause of cancer-related mortality worldwide [1–3]. The overall 5-year survival rate is only 21.7%, but it increases to 59.8% for patients at localized stages [4, 5]. Low dose computed tomography (LDCT) [6–8] is arguably efficient for early malignancy. The overdiagnosis of benign lung nodules can be dominant, which leads to unnecessary invasive needle biopsy and radiation exposures [9, 10]. This largely limits LDCT as the primary screening tool for lung cancer. Therefore, new noninvasive approaches are needed to aid early detection and nodule stratification to improve cancer diagnosis and reduce burden in healthcare.

DNA methylation changes, alongside other tumor-derived features [11, 12], become promising biomarkers for lung cancer. A recent study had discovered that cancer associated methylation abnormalities are present in patients’ blood even from asymptomatic stages, suggesting a potential noninvasive mean for cancer screening [13]. Several lines of evidence had found that common methylation alterations in ctDNA can be detected across multiple cancer types with high sensitivity and specificity [14]. However, the prediction accuracy for lung cancer was reported to be limited or contradictory, making the clinical utility difficult.

The rapid advancements in liquid biopsy revolutionize noninvasive detection of cancers, however many challenges remain. The fraction of ctDNA present in cell-free DNA from plasma is only at a level of 0.1% or below, compounding technical challenges for detecting early-stage cancers. Current sequencing-based methods involve complex procedures to handle limited ctDNA, which leads to severe sample loss and reduced analytic sensitivity. Lastly, genome-wide profiling and deep sequencing of ctDNA features in blood has been proposed as noninvasive methods in several cancer types but restricted from clinical practice regarding the labor intensity and the high running cost.

In this study, we aimed to develop an ultrasensitive and deployable PCR-based DNA methylation assay for lung cancer and assess its clinical utilities in a full spectrum of lung diseases. We profiled methylomes of lung cancer patients to identify lung cancer-specific DNA methylation markers, and then integrated the most informative targets into a simplified multi-locus qPCR assay, LunaCAM. We developed two different models to sensitively identify early-stage lung cancer and specifically stratify benign diseases. Implemented in an independent sample set, LunaCAM models were validated by classifying lung cancer from other conditions at a high degree of accuracy, demonstrating its promise for early screening and diagnostic aids for lung cancer.

## Methods

### Study design and sample characteristics

Tissue and blood samples were collected at West China Hospital and Chengdu ShangJin NanFu Hospital in China from 2015 to 2021. Eligible patients had blood drawn prior to disease-related treatment or resection. Sample classification and staging of lung nodules were confirmed by histopathological examination. Cancer was staged according to the TNM system of the American Joint Committee on Cancer (8th edition). Benign nodules were defined as hamartoma, bronchiolar adenoma, tuberculosis, granulomatous inflammation and chronic inflammation nodules according to WHO classification of tumor (5th edition): Thoracic Tumor [15]. Benign pulmonary diseases include pneumonia, asthma, COPD, pulmonary interstitial disease, and chronic inflammation disease [16–18]. Standard algorithms for the management of pulmonary nodules, including the Fleischner Society pulmonary nodule recommendations, were used to determine clinical management.

### Sample size for adequate sensitivity/specificity

The formula for sample size estimation with (1-α) % confidence level is driven as follows:

$$ n= \frac{{{(Z}_{1-\alpha /2})}^{2}P(1-P)}{{d}^{2}}$$

where *P* is pre-determined value of sensitivity (or specificity); ${Z}_{1-\alpha /2}$ is the z-value for standard normal distribution with left-tail probability (1-α/2); and d is one half the desired width of the confidence interval. This study was designed to test a sensitivity/specificity of 90% with 95% confidence and maximum marginal error of 0.1, it would require at least 35 cancer samples and 35 healthy/benign samples in the dataset.

### Library construction and data analysis of PanSeer assay

PanSeer libraries were constructed according to the protocol described previously [13]. For each target regions, average methylation fraction was defined as the total number of methylated CpGs divided by total number of detected CpGs. In order to assess the discriminative power of selected markers on classifying lung cancer and healthy plasma samples, a logistic regression (LR) classifier was built using scikit-learn package’s LogisticRegression module in plasma training samples.

### Quantitative PCR assay

For 10-20ng cfDNA samples, an amplification phase is carried out with the primer pool with 3 min at 95 °C, followed by 8 cycles of 30 s at 95 °C and 60 s at 56 °C, using ProFlexTM PCR System (Thermo Fisher). The amplified products were then aliquoted for quantitative PCR using the standard procedure (Luna® Universal Probe qPCR Master Mix (NEB)) in ABI 7500 Real-Time PCR thermal cycler. Reference gene ACTB was used as experimental quality control.

### Construction of LunaCAM classifiers

Markers were stepwise tested and ranked by AUC to explore the best model by using LogisticRegression (python v3.8.5, scikit-learn package v0.23.2). The marker with the highest AUC was selected as the anchor marker, and the combination with the rest markers was conducted by 10 × 4-fold cross-validations for each addition. The final model with the optimized combination was determined by the highest AUC of the cross-validations.

### Statistical analysis

Mann-Whitney U test was utilized to compare the model scores among different covariant groups. Accuracy metrics were computed for each sample set and subset based on sample covariates. Sensitivity is defined as true positives/ (true positives + false negatives). Specificity is defined as true negatives/ (true negatives + false positives). PPV (positive predictive value) is calculated as the ratio of true positives to the total number of positive test results. NPV (negative predictive value) is calculated as the ratio of true negatives to the total number of negative test results. Confidence interval of AUC was calculated by 1000-times bootstrapping.

## Results

### Study design and potential clinical utilities

**Fig. 1:**
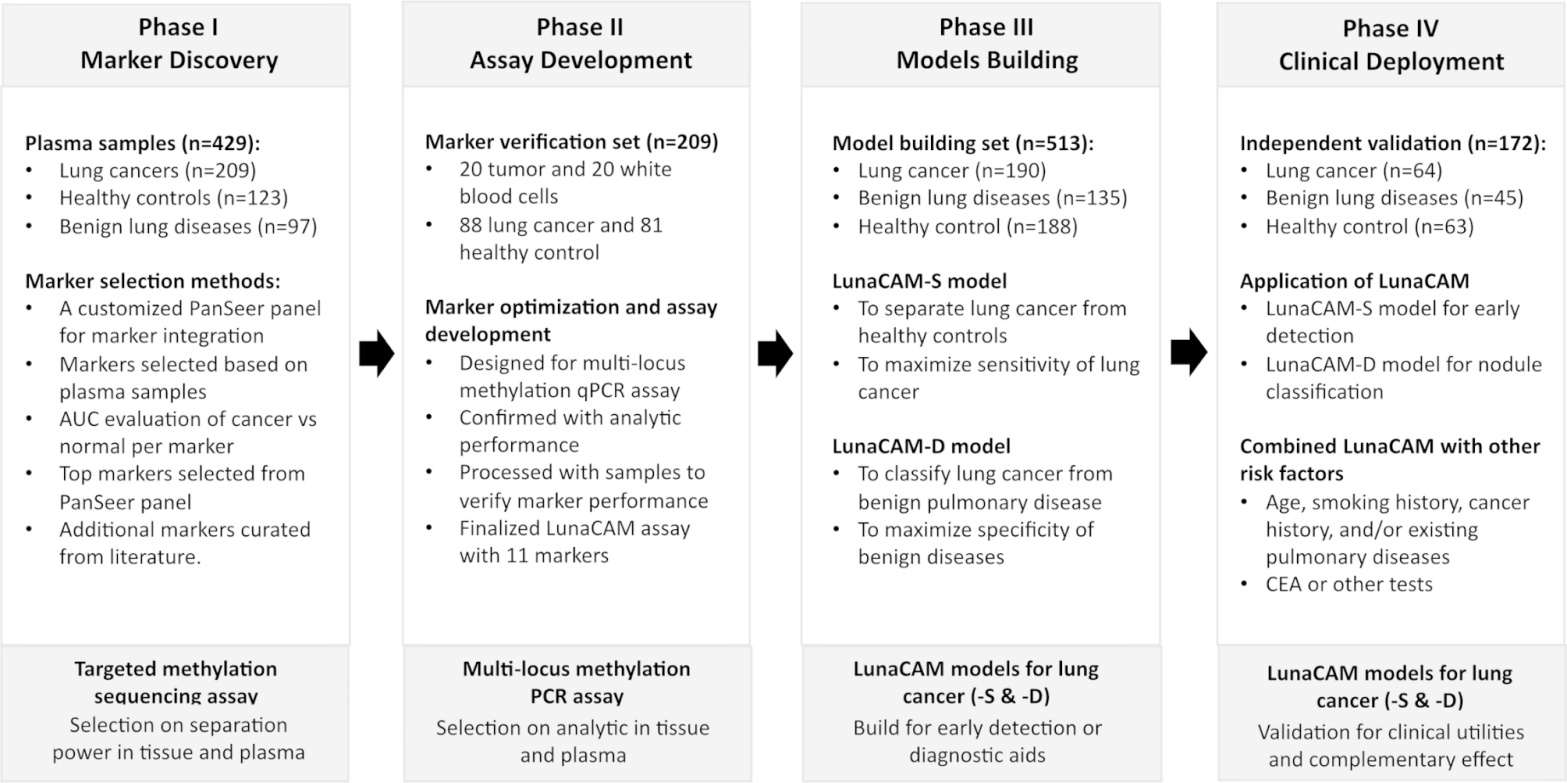
Illustration of the study design for LunaCAM model development

This study utilized a total of 1323 samples recruited from multi-centers to stepwise develop LunaCAM assay (Fig. 1, Table S1). In Phase I, we specifically utilized plasma samples to discover lung cancer markers by analyzing DNA methylation profiles of patients with lung cancer, benign diseases or no evidence of lung disease using PanSeer method [13]. In Phase II, the discriminating power of the selected markers were verified in additional tissue and plasma samples. The most informative markers were then defined to develop the multi-locus PCR assay, LunaCAM. In Phase III, different LunaCAM classifiers were built in 513 plasma samples to separate lung cancer patients from healthy individuals or patients with lung benign diseases. In Phase IV, we validated and deployed LunaCAM models to 172 plasma samples to stratify patients with lung cancer, benign diseases against healthy controls.

### Identification of DNA methylation markers to distinguish lung cancer from normal tissues

We sought to screen methylation markers specific for lung cancer which efficiently present in blood ctDNA. A previously published targeted bisulfite sequencing approach, PanSeer, was chosen to integrate a set of differentially methylated regions (DMR) for lung cancer (see eMethods). Those DMRs were defined based on publicly available microarray, whole genome bisulfite sequencing data from The Cancer Genome Atlas (TCGA) and internal reduced representation bisulfite sequencing data. A total of 165 DMRs was designed in a PanSeer panel to validate their robustness in blood (Table S2).

**Fig. 2:**
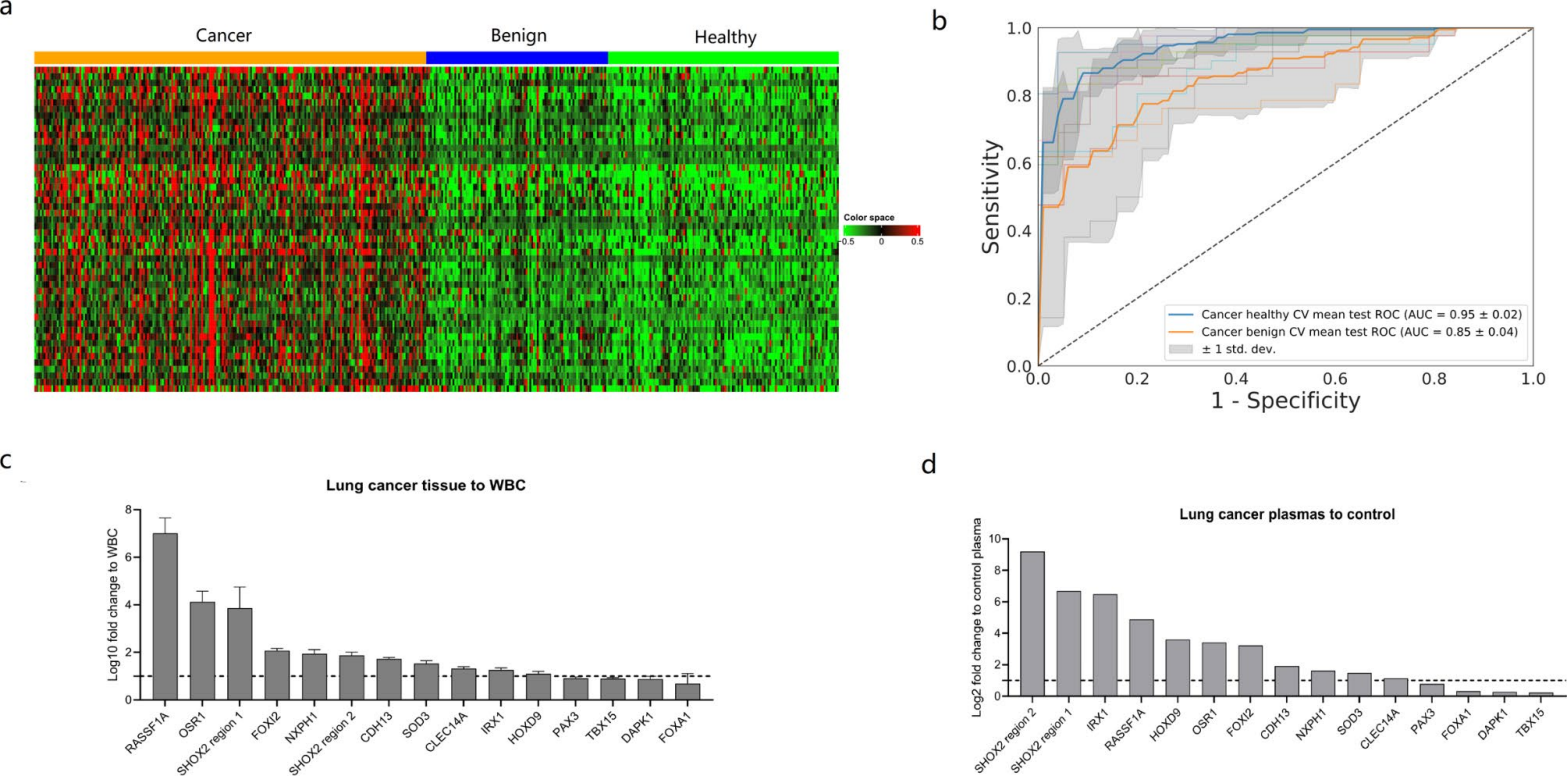
LunaCAM markers selection and optimization: (**A**) Heatmap of top 50 markers in plasma (PanSeer); (**B**) Cross-validation ROC curves based on selected markers in plasma (PanSeer); (**C**) Verification of selected markers in tissue and plasma by PCR assay

Selected DMRs were specifically examined in plasma samples derived from a variety of lung diseases to ensure their efficacy in blood. We processed a total of 429 plasma samples including 209 patients with lung cancer, 97 patients with benign lung nodules and 123 healthy individuals by using PanSeer panel with roughly 25 million sequencing reads per library. After filtering out low quality reads, regions with at least 100X coverage were used for downstream analysis. We selected DMR markers that have discriminating power between healthy individuals and cancer patients with fold change and statistical significance (see Methods). For cancer-specific hypermethylated markers, we added a filtering criterion requiring that these markers have low methylation in healthy human plasma samples. DMRs were ranked with the separation power distinguishing lung cancer from other types in addition to the fold change of the methylation level distribution, which led to the top 50 markers selected with statistical significance (Fig. 2A, Table S2). These selected DMRs were able to separate lung cancers from healthy controls and benign nodules with AUC of 0.95 and 0.85, respectively (Fig. 2B).

### LunaCAM assay development

We sought to further optimize the methylation marker candidates to the size that is feasible to implement with multiplexed qPCR. Analysis of GO terms and literature curation for markers focus on those associated with carcinogenesis, DNA binding and regulatory factor activity. In addition, we took a conservative approach to remove markers that are not located in the proximal regions of a gene or difficult for qPCR primer design. Overall, we focused on 15 DMR markers including multiple well documented genes associated with lung oncogenesis, such as SHOX2 and HOX family genes (Table S2).

To verify the feasibility of these selected markers, we examined the marker methylation status in a collection of 20 lung tumor and 20 white blood cell (WBC) samples with qPCR. The candidates were ranked by a pattern that we considered suitable for lung cancer detection in plasma: a higher signal in tumor and a lower background in blood (Fig. 2C). We also evaluated these targets with 169 plasma samples of 88 lung cancer and 81 healthy individuals to confirm their performance (Fig. 2D). Eventually, 11 markers having higher detection power in plasmas (log_2_(fold change _cancer vs. healthy_) > 1, *p* value < 0.05) and lower background in WBC (log_10_(fold change _tumor vs. WBC_) > 1) were selected for a multi-locus blood test, designated as LunaCAM (see Methods). We also estimated the methylation level of each CpG sites in these 11 markers using targeted methylation sequencing data in Phase I. We observed higher methylation levels of these CpG sites in cancer samples than healthy/benign samples (Figure S1), which further verified the feasibility of these markers.

### LunaCAM model building

To establish classifiers of LunaCAM assay, we conducted a case-control study using samples collected from multiple hospitals across a variety of lung diseases. In total, we collected blood samples from 190 patients with lung cancer, 135 patients with lung benign diseases including benign nodules, chronic pneumonia, or infection and 188 healthy individuals with no nodule findings with LDCT (Table S1). The patients were intentionally weighted to early stages of lung cancer, which 41.0% of them were diagnosed at stage I with the median tumor size of 2 cm. All plasma samples were processed with the LunaCAM assay reporting the methylation levels for each marker.

Given that preference for sensitivity and specificity is applied for different clinical uses, we intended to develop two models: LunaCAM-S for early detection in average-risk population, and LunaCAM-D for nodule classification in higher-risk population. The sample set with a full spectrum of lung diseases allows us to utilize different combinations for different model trainings, followed by model testing in the untouched categories. Model building and robustness assessment were conducted and estimated by 10 × 4-fold cross-validation, which resulted in a score for each one by averaging the cross-validation repeats (see Methods). The LunaCAM-S classifier was designed to maximize the sensitivity for detecting lung cancer, which achieved an average AUC of 0.90 in the training set and detected 91.6% of lung cancer patients (Figure S2A, Table 1). The performance of this model was partially confirmed, by which the benign individuals was separate from cancers with an AUC of 0.73 (Figure S2B). On the contrary, The LunaCAM-D classifier was intended to stratify lung cancer from benign diseases, which required a high specificity with an average AUC of 0.81 (Figure S2C). As expected, this model showed a similar power to distinguish healthy from cancer with an AUC of 0.79 (Figure S2D). Table S3 summarizes the details of 11 markers, out of which 7 were used in LunaCAM-S and 6 in LunaCAM-D. Two markers (Marker3, Marker4) associated with the gene SHOX2 were used in both models. These classifiers were then locked in additional validation set.

**Table 1:** Performance metrics of LunaCAM models in plasma samples

	LunaCAM-S model						LunaCAM-D model				
		Total	Negative	Positive	Sensitivity (95% CI)	Specificity (95% CI)	Total	Negative	Positive	Sensitivity (95% CI)	Specificity (95% CI)
Plasma (model building set)											
	Cancer	190 (training)	16	174	91.6%(90.1–93.4%)	-	190 (training)	83	107	56.3%(52.6–59.9%)	-
	Benign	135 (test)	57	78	-	42.2%(38.0-46.3%)	135 (training)	132	3	-	97.8%(97.2–99.1%)
	Health	188 (training)	113	75	-	60.1%(56.7–63.3%)	188 (test)	166	22	-	88.3%(86.0-90.7%)
**Plasma (Independent validation set)**											
	Cancer	64	6	58	90.6%(88.2–94.1%)	-	64	29	33	51.6%(45.1–56.9%)	-
	Benign	45	17	28	-	37.8%(30.6–44.4%)	45	42	3	-	93.3%(91.7–97.2%)
	Health	63	39	24	-	61.9%(56.0–68.0%)	63	53	10	-	84.1%(80.0–88.0%)
**Plasma (Total)**											
	Cancer	254					254				
	I	108	14	94	87.0%(83.7–90.7%)	-	108	55	53	49.1%(44.2–53.5%)	-
	II	38	2	36	94.7%(93.3–100.0%)	-	38	15	23	60.5%(53.3–70.0%)	-
	III/IV	108	6	102	94.4%(93.0-96.5%)	-	108	44	64	59.3%(54.7–64.0%)	-
	Health	251	152	99	-	60.6%(57.5–63.5%)	251	219	32	-	87.3%(85.5–89.5%)
	Benign	180	74	106	-	41.1%(37.5–44.4%)	180	174	6	-	96.7%(95.8–97.9%)

### Validation of LunaCAM for early detection and classification of lung cancer

To evaluate the predictive performance of LunaCAM models, we deployed LunaCAM test in an independent group of individuals from multiple centers. The subjects in the collection included symptomatic ones with pulmonary related symptoms, such as cough or dyspnea, and asymptomatic ones at general health checks. All subjects performed a LDCT at the time of the clinic visit to assess pulmonary diseases with or without a finding of lung nodules. Among these individuals, 64 were diagnosed as lung cancer after biopsy or surgery, 45 with histologically proven benign nodules or common benign pulmonary diseases, and 63 having no evidence of lung diseases (Fig. 1).

**Fig. 3:**
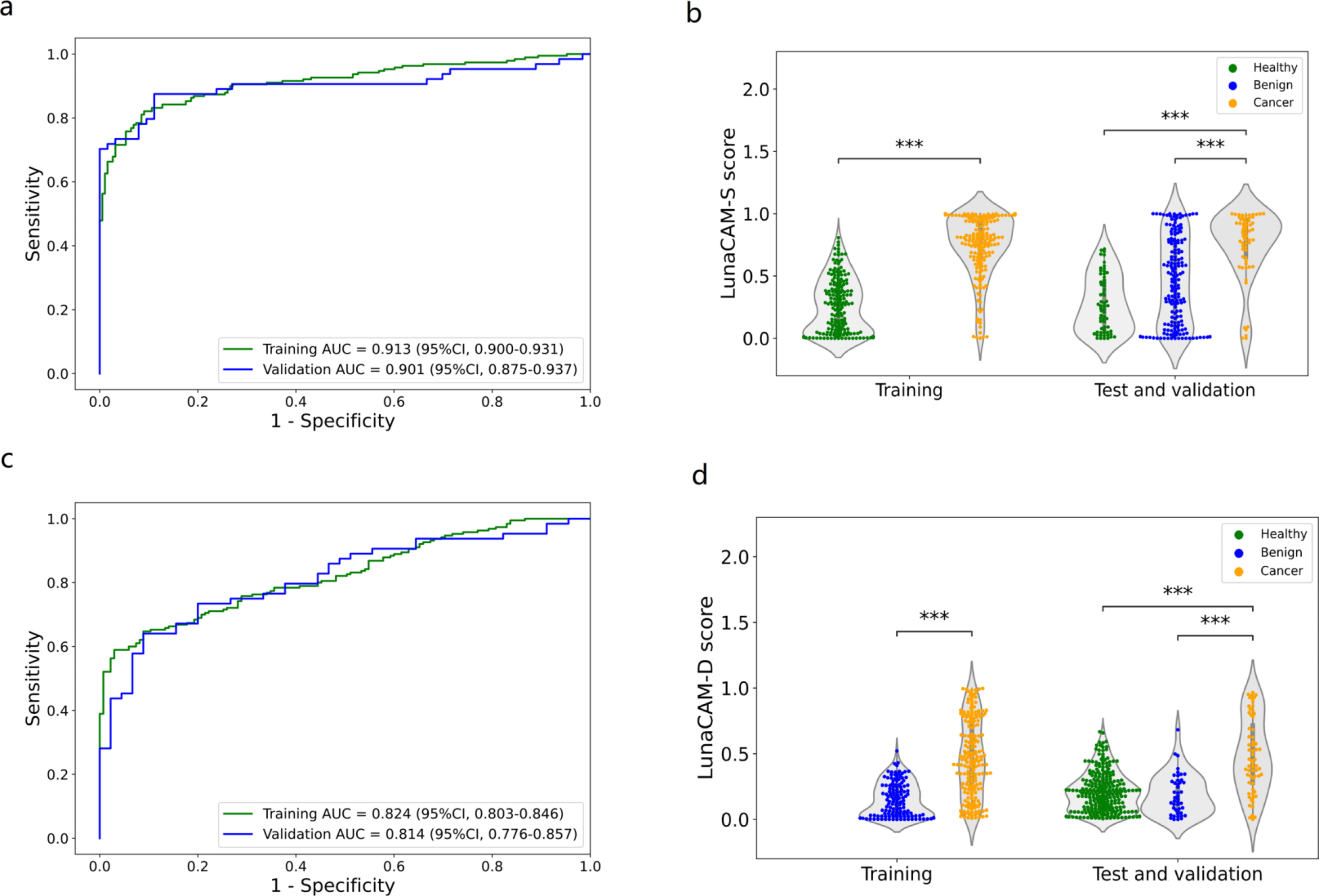
LunaCAM Model building and validation: (**A**) ROC curves of LunaCAM-S in training and validation; (**B**) LunaCAM-S score distribution in training, test and validation; (**C**) ROC curves of LunaCAM-D in training and validation; (**D**) LunaCAM-D score distribution in training, test and validation

**Fig. 4:**
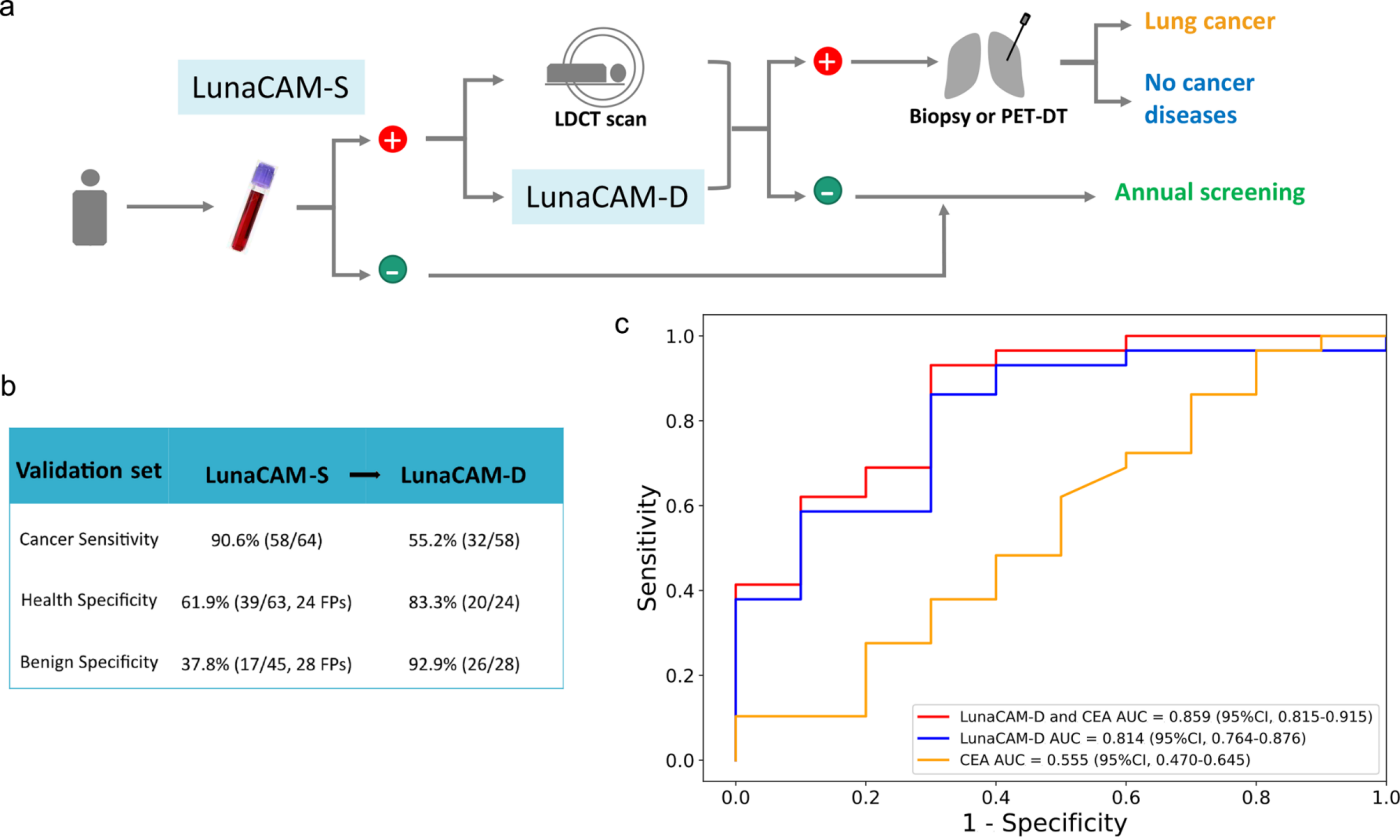
LuncaCAM models for potential implementation: (**A**) Schematic representation of the proposed implementation LunaCAM models into the current standard practice for lung cancer; (**B**) Summary of combined model in validation set; (**C**) ROC curves of CEA, LunaCAM-D, and the combined model of LunaCAM-D and CEA.

All blood samples were processed in a blinded manner to evaluate LunaCAM assay performance. We first predicted the cancer status for individuals according to whether the LunaCAM-S score was above or below the cutoff. Similar sensitives and specificities were obtained in this validation set with an AUC of 0.90 for lung cancer compared to healthy control, which identified 90.6% of lung cancer patients (Fig. 3A; Table 1). Consistently, this model also exhibited certain level of discrimination for benign diseases, suggesting that LunaCAM approach is generalizable across different lung cancer cohorts (Fig. 3B; Table 1). LunaCAM-D classifier was next validated to stratify lung cancer from pulmonary benign diseases. LunaCAM-D was able to reproduce a consistent AUC of 0.81 in validation, and a similar separation for healthy from lung cancer with an AUC of 0.76 (Fig. 3C and D, Figure S2D). When LunaCAM-D was applied to LunaCAM-S positive cases as in practice, 83.3% of patients miscalled by LunaCAM-S was corrected while 55.2% lung cancer patients maintained positive (Fig. 4A and B). With the combination of LunaCAM-S and LunaCAM-D, the sensitivity of cancer can reach 50% at a high specificity of 94.4%. In the validation set of the current study, the PPV was 84.2% and NPV was 76.1%. For an intended-use population with a prevalence of cancer at 10% [19], the PPV was calculated to be 46.1% and the PPV was as high as 94.6%.

### Covariant analysis and clinical utility of LunaCAM

We next investigated if demographic or clinic characteristics were associated with LunaCAM scores (Table S4). No correlations were observed between LunaCAM scores and age, gender or smoking history (Figure S3 and S4). No significant difference was observed in cancer patients with different stages, suggesting LunaCAM models might capture cancer signals from early-stages (Figure S5A and S5B, Table 1). In the combined set of all plasma samples, LunaCAM-S model has a sensitivity of up to 87.0% for stage I cancer, while the LunaCAM-D model can achieve a sensitivity of 49.1% for stage I cancer at high specificity levels of 96.7% for benign samples and 87.3% for healthy samples (Table 1). Similar result was observed between LunaCAM scores with tumor size (Figure S5C and S5D). LunaCAM-S model detected 86% (12 of 14) tumors less than nodule diameter of 1.2 cm equivalent to 1 cm^3^ tumor [20], suggesting a superior sensitivity of LunaCAM-S for even relatively small tumors. Histologic analysis of lung cancer revealed that squamous cell, small cell and large cell lung cancer effectively separated from healthy or benign samples via LunaCAM models, consistent with adenocarcinoma lung cancers (Figure S5E and S5F).

Lastly, we sought to explore the combination of LunaCAM-D with risk factors known for lung and other cancers in a multi-modal manner. Among all factors, CEA showed a higher level in cancer patients than those without cancer, but it underperforms LunaCAM-D model across all stages (AUC_CEA_ = 0.56, AUC_LunaCAM-D_ = 0.81) (Fig. 4C). However, the combination of LunaCAM-D and CEA revealed an even higher overall AUC of 0.86, especially leading a significant increase in sensitivity when specificity is higher. Although limited by the sample size, this analysis does suggest that the combination of LunaCAM-D with serum protein may improve nodule classification compared to those tests alone.

## Discussion

In this study, we described the development, validation, and implementation of LunaCAM assay targeting ctDNA methylation of lung cancer. Through multiple rounds of verification, we integrated lung cancer methylation markers into facile qPCR procedure, LunaCAM. The LunaCAM models were validated in an independent sample cohort to test the generalization. LunaCAM-S model achieved a superior sensitivity of lung cancer, which may improve the accessibility of noninvasive screening for lung cancer. LunaCAM-D model, on the other hand, was stringent in specificity to aid for diagnostics and prevent overtreatment. The two models can be sequentially applied for different utilities and possible combination with other standard procedures. Altogether, we demonstrated that LunaCAM could provide a reliable and cost-effective diagnostic aid for lung cancer and the combinatory multi-factor analysis might further increase the accuracy of the approach.

Efficacy of LDCT screening for lung cancer has been under debate for decades regarding patient compliance, complex infrastructure, and repetitive radiation exposure [21, 22]. Although screening high-risk population with LDCT is proposed to reduce lung cancer mortality [6, 7], the percentage of eligible smokers under annual LDCT screening remains exceptionally low [23]. Enormous efforts have been made for searching of molecular biomarkers to facilitate the lung cancer screening [24]. Several promising molecular candidates have been discovered and reported including autoantibodies [25], microRNAs [26, 27], ctDNA mutation [28–30], genome fragmentomes [12] and DNA methylation [31]. The accuracy of the prediction of lung cancer was relatively low through assessment of the levels of proteins and ctDNA mutations [32, 33]. The methylation profiling of ctDNA in blood has becoming appealing source of innovative cancer biomarkers recently [34–36]. The circulating cell-free genome atlas (CCGA) study has shown that ctDNA methylation patterns outperformed genome-wide fragmentome and ctDNA variants [37]. Although ctDNA methylation signatures are informative, the detection sensitivity of lung cancer, especially for stage I, was dropped down to 21.9% in a recent report of CCGA study [14]. Thus, challenges in the field of biomarker discovery for lung cancer remain to meet the clinical needs.

Despite the rapid development of molecular methodologies, biomarker tests of lung cancer are rarely used in routine clinical practice. Cost-effectiveness, test logistics, and simplified procedure are important factors to consider in clinics [38, 39]. NGS-based liquid biopsy assays were mainly utilized in biomarker discovery studies; however, the high running cost and complex procedures limit its applicability as screening tests. In contract, LunaCAM was optimized from NGS-based assay, which ensures a high performance and a simple PCR-based operation. In addition, two LunaCAM models were adjusted to enable robust performance in different intended uses. Altogether, these features of LunaCAM assay maximize its values in the current standard of care as cost effective and viable aid.

There are several limitations in this study. The performance of LunaCAM models need to eventually validate in prospective trials. Incomplete demographic and clinical data, especially the smoking/cancer history in healthy group, led to limited multivariable analysis with risk factors. Lastly, follow-up studies would be informative for those positive in LunaCAM but currently having no evidence of lung cancer.

## Conclusions

LunaCAM provides a simple and inexpensive blood test to refine risk assessment of lung cancer prior to LDCT and to characterize pulmonary disorders alongside standard practice. It has the potential to broaden access for lung cancer screening, which could efficiently identify patients at higher risk of lung cancer when deployed in clinic.

## Electronic supplementary material

Below is the link to the electronic supplementary material.

Supplementary Material 1

## List of abbreviations

**ctDNA**: Circulating tumor DNA
**LunaCAM**: Lung cancer alertness by ctDNA methylation.
**AUC**: Area under curve
**PCR**: Polymerase chain reaction
**CEA**: Carcinoembryonic antigen
**LDCT**: Low dose computed tomography
**DMR**: Differentially methylated regions
