# Supplementary material for: Early detection and stratification of lung cancer aided by a cost-effective assay targeting circulating tumor DNA (ctDNA) methylation
Source: Respir Res. 2023 Jun 17;24:163. doi: 10.1186/s12931-023-02449-8 (PMC10276518; doi:10.1186/s12931-023-02449-8)
Supplement: Supplementary file 1 — Supplementary Material 1 [file 12931_2023_2449_MOESM1_ESM.docx]

Supplementary Materials for

**Early detection and stratification of lung cancer aided by a cost-effective assay targeting circulating tumor DNA (ctDNA) methylation**

**The following sections include:**

Supplementary Table S1. Sample compositing for each phase.

Supplementary Table S2. Selected 165 DMRs from PanSeer data.

Supplementary Table S3. Summary of the final 11 markers.

Supplementary Table S4. Patient demographic and clinical characteristics.

Supplementary Figure S1. Heatmap of methylation levels of CpG sites in the final 11 markers in Phase I plasma samples, using targeted methylation sequencing data generated from PanSeer assay.

Supplementary Figure S2. LunaCAM model building and validation.

Supplementary Figure S3. Covariant analysis of LunaCAM-S scores against gender, smoking history, and age.

Supplementary Figure S4. Covariant analysis of LunaCAM-D scores against gender, smoking history, and age.

Supplementary Figure S5. Correlation of LunaCAM scores with clinical characteristics.

Supplementary Table S1. Sample compositing for each phase.

|  | **Phase I** | **Phase II** | | **Phase III** | **Phase IV** |
| --- | --- | --- | --- | --- | --- |
| Assay | PanSeer | LunaCAM | | LunaCAM | |
| Sample type |  |  |  |  |  |
| Tissue |  | 40 |  |  |  |
| Plasma | 429 |  | 169 | 513 | 172 |
| Subject age |  |  |  |  |  |
| Median  (min~max) | 54  (24~83) | 59  (46~79) | 55  (21~78) | 55  (23~93) | 57  (23~88) |
| Gender |  |  |  |  |  |
| Male | 234 | 10 | 82 | 254 | 87 |
| Female | 195 | 10 | 87 | 259 | 85 |
| Diagnosis |  |  |  |  |  |
| Cancer | 209 | 20 | 55 | 190 | 64 |
| Benign* | 97 |  | 26 | 135 | 45 |
| Normal | 123 | 20 | 88 | 188 | 63 |
| Cancer Stage |  |  |  |  |  |
| I | 142 | 10 | 30 | 78 | 30 |
| II | 28 | 6 | 23 | 25 | 13 |
| III/IV | 28 | 4 | 35 | 87 | 21 |
| NA | 11 |  |  |  |  |

* Benign pulmonary diseases include benign nodules, chronic pneumonia, lung infections, pulmonary bullae, fibrous foci and calcification

Supplementary Table S2. Selected 165 DMRs from PanSeer data.

| Region | Gene | AUC | Rank |
| --- | --- | --- | --- |
| chr14:57275744:57276023 | OTX2 | 0.900478469 | 0 |
| chr2:73147693:73147893 | EMX1 | 0.892857143 | 1 |
| chr14:38724555:38725035* | CLEC14A | 0.888835726 | 2 |
| chr3:157812131:157812482* | SHOX2-region1 | 0.882105263 | 3 |
| chr13:53421052:53421252 | PCDH8 | 0.870972887 | 4 |
| chr2:71115853:71116351 | VAX2 | 0.870940989 | 5 |
| chr2:176945337:176945719 | EVX2 | 0.863125997 | 6 |
| chr4:24801639:24802072* | SOD3 | 0.860829346 | 7 |
| chr6:137814694:137814894 | OLIG3 | 0.858277512 | 8 |
| chr7:8482114:8482413* | NXPH1 | 0.85738437 | 9 |
| chr2:176987322:176987856* | HOXD9 | 0.856204147 | 10 |
| chr2:223163219:223163595* | PAX3 | 0.84953748 | 11 |
| chr19:19650947:19651147 | CILP2 | 0.84937799 | 12 |
| chr5:3599720:3599934* | IRX1 | 0.846953748 | 13 |
| chr1:63785793:63786090 | FOXD3 | 0.846443381 | 14 |
| chr6:154360602:154360817 | OPRM1 | 0.841275917 | 15 |
| chr13:112717238:112717438 | SOX1 | 0.828953301 | 16 |
| chr2:162280397:162280666 | TBR1 | 0.822440868 | 17 |
| chr14:57265398:57265598 | OTX2 | 0.818213716 | 18 |
| chr3:157821224:157821604* | SHOX2-region2 | 0.818086124 | 19 |
| chr11:44326261:44326490 | ALX4 | 0.816810207 | 20 |
| chr8:55370821:55371074 | SOX17 | 0.816623652 | 21 |
| chr5:115152406:115152637 | CDO1 | 0.812376396 | 22 |
| chr16:82660460:82660774* | CDH13 | 0.81122807 | 23 |
| chr1:50884536:50884747 | DMRTA2 | 0.807591707 | 24 |
| chr16:51189898:51190257 | SALL1 | 0.806092504 | 25 |
| chr10:133110769:133110969 | PPP2R2D | 0.805741627 | 26 |
| chr11:125036402:125036613 | PKNOX2 | 0.805550239 | 27 |
| chr4:154709519:154709799 | SFRP2 | 0.804880383 | 28 |
| chr5:178421448:178421697 | GRM6 | 0.803508772 | 29 |
| chr2:176969332:176969729 | HOXD11 | 0.803253589 | 30 |
| chr2:5833431:5833666 | SOX11 | 0.799362041 | 31 |
| chr4:8863209:8863434 | HMX1 | 0.79738437 | 32 |
| chr1:119535692:119536030* | TBX15 | 0.796778309 | 33 |
| chr10:131770965:131771313 | EBF3 | 0.795215311 | 34 |
| chr14:38061327:38061706* | FOXA1 | 0.794896332 | 35 |
| chr2:5832783:5833091 | SOX11 | 0.791738437 | 36 |
| chr12:33592774:33592974 | SYT10 | 0.790526316 | 37 |
| chr7:152622494:152622712 | ACTR3B | 0.78953748 | 38 |
| chr15:68114350:68114550 | SKOR1 | 0.788580542 | 39 |
| chr15:27113022:27113477 | GABRA5 | 0.788325359 | 40 |
| chr10:108924091:108924291 | SORCS1 | 0.786889952 | 41 |
| chr5:3602162:3602362 | IRX1 | 0.784082935 | 42 |
| chr11:636862:637062 | DRD4 | 0.783923445 | 43 |
| chr8:55370383:55370609 | SOX17 | 0.782264753 | 44 |
| chr2:239755167:239755412 | TWIST2 | 0.78108453 | 45 |
| chr10:129534694:129534935* | FOXI2 | 0.778724083 | 46 |
| chr6:19691753:19691953 | ID4 | 0.778660287 | 47 |
| chr5:134870613:134870990 | NEUROG1 | 0.778213716 | 48 |
| chr10:118892523:118892723 | VAX1 | 0.777464115 | 49 |
| chr11:31837311:31837611 | PAX6 | 0.776044657 | 50 |
| chr1:119527250:119527450 | TBX15 | 0.77014139 | 51 |
| chr1:50884300:50884500 | DMRTA2 | 0.768931419 | 52 |
| chr5:16179948:16180148 | MARCH11 | 0.765933014 | 53 |
| chr11:58672824:58673024 | GLYATL1 | 0.765550239 | 54 |
| chr10:8097474:8097837 | GATA3 | 0.762838915 | 55 |
| chr10:110672177:110672377 | XPNPEP1 | 0.762360447 | 56 |
| chr2:177025034:177025300 | HOXD4 | 0.760414673 | 57 |
| chr5:3606438:3606670 | IRX1 | 0.759457735 | 58 |
| chr5:178770871:178771144 | ADAMTS2 | 0.759266348 | 59 |
| chr8:686927:687127 | TDRP | 0.75891547 | 60 |
| chr14:37126872:37127078 | PAX9 | 0.757192982 | 61 |
| chr1:119526811:119527011 | TBX15 | 0.756427432 | 62 |
| chr8:25907762:25907983 | EBF2 | 0.756236045 | 63 |
| chr9:126778279:126778644 | LHX2 | 0.756172249 | 64 |
| chr16:22825689:22825889 | HS3ST2 | 0.754800638 | 65 |
| chr8:23564023:23564306 | NKX2-6 | 0.754385965 | 66 |
| chr13:112758741:112758954 | SOX1 | 0.753269537 | 67 |
| chr7:27244589:27244849 | HOXA13 | 0.753269537 | 68 |
| chr1:237205919:237206212 | RYR2 | 0.752791069 | 69 |
| chr2:111876461:111876753 | BCL2L11 | 0.751812953 | 70 |
| chr10:22542122:22542322 | COMMD3 | 0.750558214 | 71 |
| chr3:181444089:181444289 | SOX2 | 0.748803828 | 72 |
| chr19:51520252:51520452 | KLK10 | 0.747863248 | 73 |
| chr1:156611838:156612099 | BCAN | 0.745837321 | 74 |
| chr17:48042351:48042687 | DLX4 | 0.743891547 | 75 |
| chr16:31580122:31580353 | AHSP | 0.741977671 | 76 |
| chr5:1876269:1876469 | IRX4 | 0.740669856 | 77 |
| chr7:27206030:27206230 | HOXA9 | 0.740510367 | 78 |
| chr17:35165517:35165717 | LHX1 | 0.74 | 79 |
| chr12:113917423:113917666 | LHX5 | 0.736969697 | 80 |
| chr3:192126117:192126324 | FGF12 | 0.736140351 | 81 |
| chr7:27204968:27205178 | HOXA9 | 0.735913462 | 82 |
| chr1:50881931:50882297 | DMRTA2 | 0.734322169 | 83 |
| chr16:22825962:22826162 | HS3ST2 | 0.732440191 | 84 |
| chr6:85476974:85477296 | TBX18 | 0.731770335 | 85 |
| chr14:37116133:37116488 | PAX9 | 0.731483254 | 86 |
| chr1:119532703:119532988 | TBX15 | 0.730877193 | 87 |
| chr2:176936260:176936480 | EVX2 | 0.729952153 | 88 |
| chr17:46666849:46667088 | HOXB5 | 0.728548644 | 89 |
| chr10:23480625:23480825 | PTF1A | 0.727751196 | 90 |
| chr6:10417560:10417760 | TFAP2A | 0.726698565 | 91 |
| chr10:7449719:7449919 | SFMBT2 | 0.726570973 | 92 |
| chr10:23481046:23481246 | PTF1A | 0.726028708 | 93 |
| chr1:151811354:151811554 | C2CD4D | 0.724274322 | 94 |
| chr2:19556785:19556985* | OSR1 | 0.723839536 | 95 |
| chr8:65282197:65282431 | BHLHE22 | 0.722902711 | 96 |
| chr4:57521292:57521580 | HOPX | 0.722870813 | 97 |
| chr2:177024578:177024778 | HOXD4 | 0.722232855 | 98 |
| chr2:177024290:177024490 | HOXD4 | 0.72169059 | 99 |
| chr2:177036795:177036995 | HOXD1 | 0.720797448 | 100 |
| chr2:177023006:177023206 | HOXD4 | 0.720063796 | 101 |
| chr8:70981887:70982121 | PRDM14 | 0.719808612 | 102 |
| chr7:156798388:156798588 | MNX1 | 0.717767145 | 103 |
| chr17:36666092:36666292 | ARHGAP23 | 0.71754386 | 104 |
| chr18:49867020:49867262 | DCC | 0.716012759 | 105 |
| chr5:140892824:140893033 | PCDHGC5 | 0.714960128 | 106 |
| chr2:468096:468344 | FAM150B | 0.710398724 | 107 |
| chr1:145562746:145563122 | PIAS3 | 0.708676236 | 108 |
| chr6:50818154:50818354 | TFAP2B | 0.708102073 | 109 |
| chr4:190940255:190940455 | FRG2 | 0.706379585 | 110 |
| chr3:25469781:25470075 | RARB | 0.703600339 | 111 |
| chr5:40681817:40682084 | PTGER4 | 0.702711324 | 112 |
| chr2:193059315:193059515 | TMEFF2 | 0.69923445 | 113 |
| chr20:55202107:55202408 | TFAP2C | 0.697480064 | 114 |
| chr7:150655260:150655562 | KCNH2 | 0.697033493 | 115 |
| chr12:30354393:30354624 | TMTC1 | 0.696937799 | 116 |
| chr13:25320388:25320680 | RNF17 | 0.695342903 | 117 |
| chr9:21994117:21994434 | CDKN2B | 0.694386694 | 118 |
| chr10:119292117:119292490 | EMX2 | 0.694130781 | 119 |
| chr1:933461:933661 | HES4 | 0.691578947 | 120 |
| chr15:29395897:29396097 | NDNL2 | 0.691578947 | 121 |
| chr8:21647523:21647723 | GFRA2 | 0.690366826 | 122 |
| chr9:19788555:19788755 | SLC24A2 | 0.687591707 | 123 |
| chr9:19789033:19789233 | SLC24A2 | 0.685662596 | 124 |
| chr6:106429583:106429783 | PRDM1 | 0.683317384 | 125 |
| chr3:38080591:38080907 | DLEC1 | 0.680223285 | 126 |
| chr10:130084908:130085108 | MKI67 | 0.679681021 | 127 |
| chr12:52311647:52311991 | ACVRL1 | 0.679457735 | 128 |
| chr5:2749400:2749600 | IRX2 | 0.676012759 | 129 |
| chr8:41166789:41167017 | SFRP1 | 0.672297297 | 130 |
| chr8:124173191:124173417 | FAM83A | 0.671547049 | 131 |
| chr10:119295843:119296149 | EMX2 | 0.668165869 | 132 |
| chr9:21974601:21974979 | CDKN2A | 0.668133971 | 133 |
| chr11:63687058:63687359 | RCOR2 | 0.667942584 | 134 |
| chr14:52735051:52735329 | PTGDR | 0.667208931 | 135 |
| chr3:129693578:129693778 | TRH | 0.663030303 | 136 |
| chr2:176956558:176956758 | HOXD13 | 0.661403509 | 137 |
| chr9:90112714:90113085* | DAPK1 | 0.656076555 | 138 |
| chr16:23847490:23847758 | PRKCB | 0.647208931 | 139 |
| chr3:50377975:50378564* | RASSF1 | 0.645199362 | 140 |
| chr3:49459532:49459732 | AMT | 0.624593301 | 141 |
| chr1:119532992:119533192 | TBX15 | 0.612745098 | 142 |
| chr19:16394438:16394677 | KLF2 | 0.612409215 | 143 |
| chr8:145105569:145105769 | OPLAH | 0.610271132 | 144 |
| chr6:41528461:41528661 | FOXP4 | 0.609633174 | 145 |
| chr2:25499956:25500318 | DNMT3A | 0.608165869 | 146 |
| chr8:145105784:145105984 | OPLAH | 0.607208931 | 147 |
| chr5:112073279:112073623 | APC | 0.602998405 | 148 |
| chr15:41795038:41795238 | ITPKA | 0.599968102 | 149 |
| chr22:50623352:50623552 | PANX2 | 0.59954166 | 150 |
| chr12:115111826:115112463 | TBX3 | 0.598022329 | 151 |
| chr3:124860723:124861087 | SLC12A8 | 0.595151515 | 152 |
| chr7:100075176:100075453 | TSC22D4 | 0.592727273 | 153 |
| chr20:55965081:55965304 | RBM38 | 0.578628389 | 154 |
| chr16:33964856:33965077 | ENSG00000259680 | 0.577926635 | 155 |
| chr22:50987078:50987403 | KLHDC7B | 0.557958533 | 156 |
| chr10:81003018:81003261 | PPIF | 0.536658999 | 157 |
| chr16:50875086:50875366 | CYLD | 0.528725962 | 158 |
| chr13:36703325:36703631 | DCLK1 | 0.484625199 | 159 |
| chr10:131265465:131265744 | MGMT | 0.452631579 | 160 |
| chr12:25055821:25056199 | BCAT1 | NA | 161 |
| chr19:51520501:51520701 | KLK10 | NA | 163 |
| chr3:129693363:129693563 | TRH | NA | 164 |
| chr7:27205235:27205435 | HOXA9 | NA | 162 |

Notes: 15 DMRs labeled with “*” were selected.

Supplementary Table S3. Summary of the final 11 markers.

| **MarkerID** | **PCR Regions** | **Gene** | **CpG number** | **Available CpG number in PanSeer panel** | **In LunaCAM-S ?** | **In LunaCAM-D ?** |
| --- | --- | --- | --- | --- | --- | --- |
| Marker1 | chr2:176987563:176987664 | HOXD9 | 14 | 14 | No | YES |
| Marker2 | chr2:19556946:19557021 | OSR1 | 6 | 3 | YES | No |
| Marker3 | chr3:157812240:157812353 | SHOX2-region1 | 16 | 16 | YES | YES |
| Marker4 | chr3:157821339:157821429 | SHOX2-region2 | 8 | 8 | YES | YES |
| Marker5 | chr3:50378061:50378154 | RASSF1A | 14 | 14 | No | YES |
| Marker6 | chr4:24801841:24801946 | SOD3 | 9 | 9 | YES | No |
| Marker7 | chr5:3599723:3599816 | IRX1 | 9 | 9 | No | YES |
| Marker8 | chr7:8482292:8482408 | NXPH1 | 10 | 10 | YES | No |
| Marker9 | chr10:129534759:129534851 | FOXI2 | 10 | 10 | YES | No |
| Marker10 | chr14:38724649:38724719 | CLEC14A | 6 | 6 | YES | No |
| Marker11 | chr16:82660402:82660511 | CDH13 | 9 | 4 | No | YES |

Note: The PCR regions and the PanSeer panel regions are slightly different, resulting in some CpG sites of PCR regions not being covered by the PanSeer panel (Marker2 and Marker11).

Supplementary Table S4. Patient demographic and clinical characteristics.

| **Characteristics** | **Total n=685** | **Healthy n=251** | **Benign disease* n=180** | **Lung cancer n=254** | **Cancer characteristics** |  |
| --- | --- | --- | --- | --- | --- | --- |
| ***Subjects*** |  |  |  |  | ***Histology*** | n |
| Age-yr | 56 (23~93) | 51 (23~80) | 59 (23~88) | 56.5 (29~93) | Adenocarcinoma | 161 |
| Gender-no (percentage) |  |  |  |  | Squamous cell carcinoma | 23 |
| Male | 339 (49.5%) | 128 (51.0%) | 83 (46.1%) | 128 (50.4%) | Small cell | 16 |
| Female | 346 (50.5%) | 123 (49.0%) | 97 (53.9%) | 126 (49.6%) | Large cell | 3 |
| Smoking history status-no |  |  |  |  | No data | 51 |
| YES | 93 | 17 | 26 | 50 | ***Stage*** | n |
| NO | 179 | 38 | 53 | 88 | carcinoma in situ | 3 |
| No data | 413 | 196 | 101 | 116 | IA | 80 |
| ***Nodules*** |  |  |  |  | IB | 25 |
| No nodules | 251 | 251 |  |  | II | 38 |
| <10 mm | 30 |  | 4 | 26 | III | 68 |
| 10~20 mm | 50 |  | 7 | 43 | IV | 40 |
| 20~30 mm | 39 |  | 2 | 37 |  |  |
| >=30 mm | 47 |  | 3 | 44 |  |  |
| No data | 268 |  | 164 | 104 |  |  |
| * Benign pulmonary diseases include benign nodules, chronic pneumonia, lung infections, pulmonary bullae, fibrous foci and calcification | | | | | |  |

Supplementary Figure S1. Heatmap of methylation levels of CpG sites in the final 11 markers in Phase I plasma samples, using targeted methylation sequencing data generated from PanSeer assay.


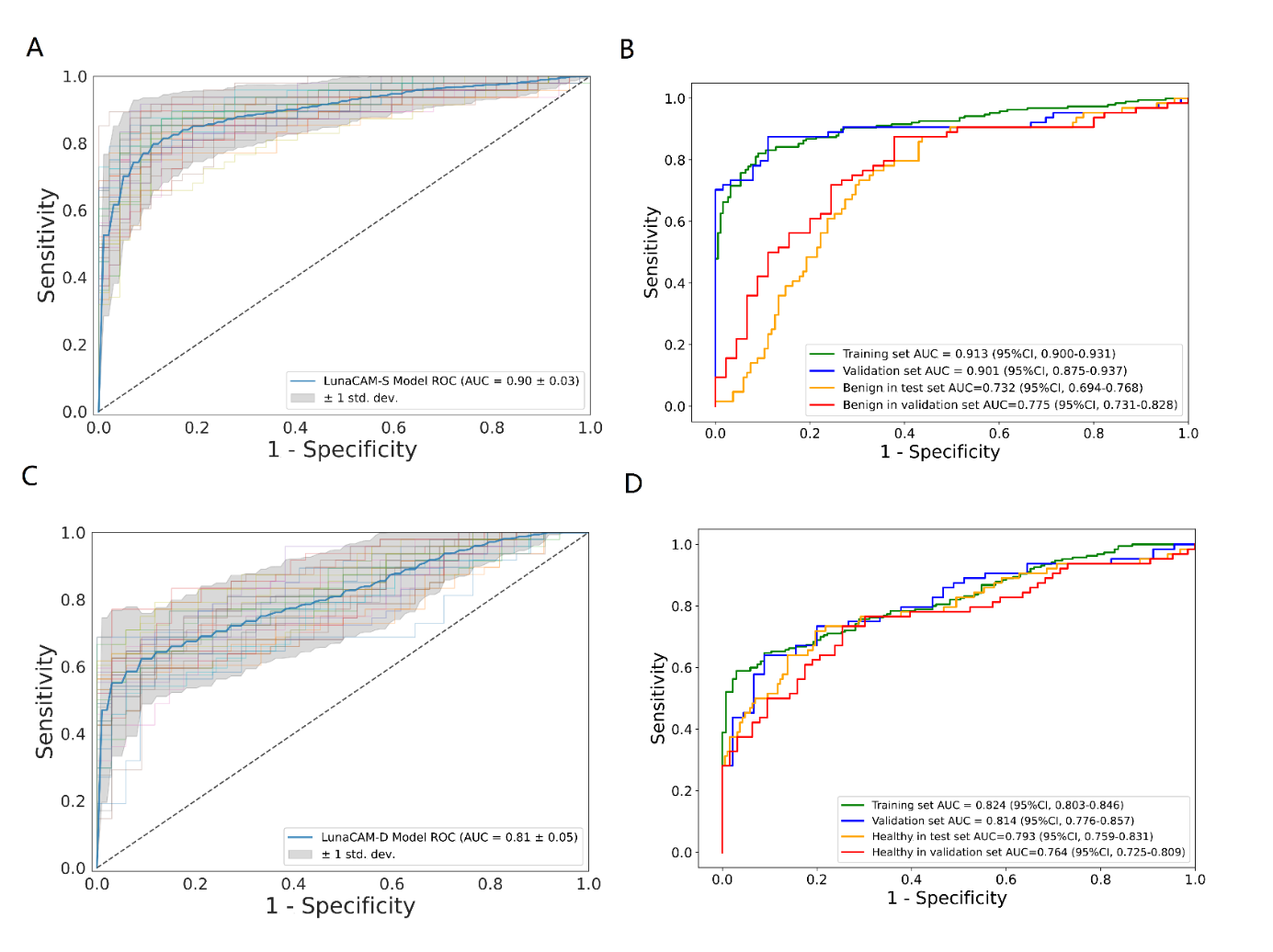


Supplementary Figure S2. LunaCAM model building and validation: A) Cross-validation ROC curves of LunaCAM-S in model building; B) training and validation ROC curves of LunaCAM-S; C) Cross-validation ROC curves of LunaCAM-D in model building; D) training and validation ROC curves of LunaCAM-D.


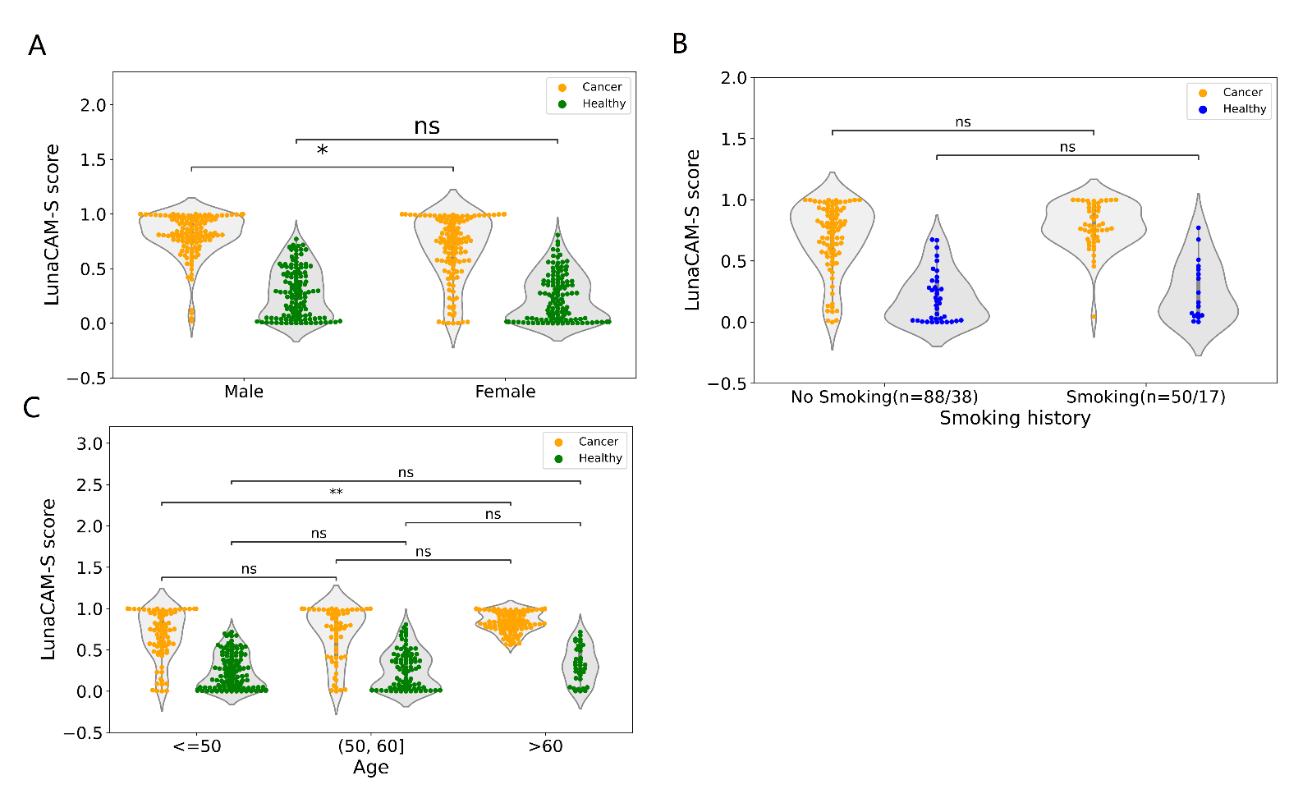


Supplementary Figure S3. Covariant analysis of LunaCAM-S scores against gender (A), smoking history (B), and age (C). Mann-Whitney u-test was used: **ns**: not significant (*p* > 0.05), *****: 0.01 < *p* <= 0.05; ******: 0.001 < *p* <= 0.01; *******: 0.0001 < *p* <= 0.001; ********: *p* < 0.0001.


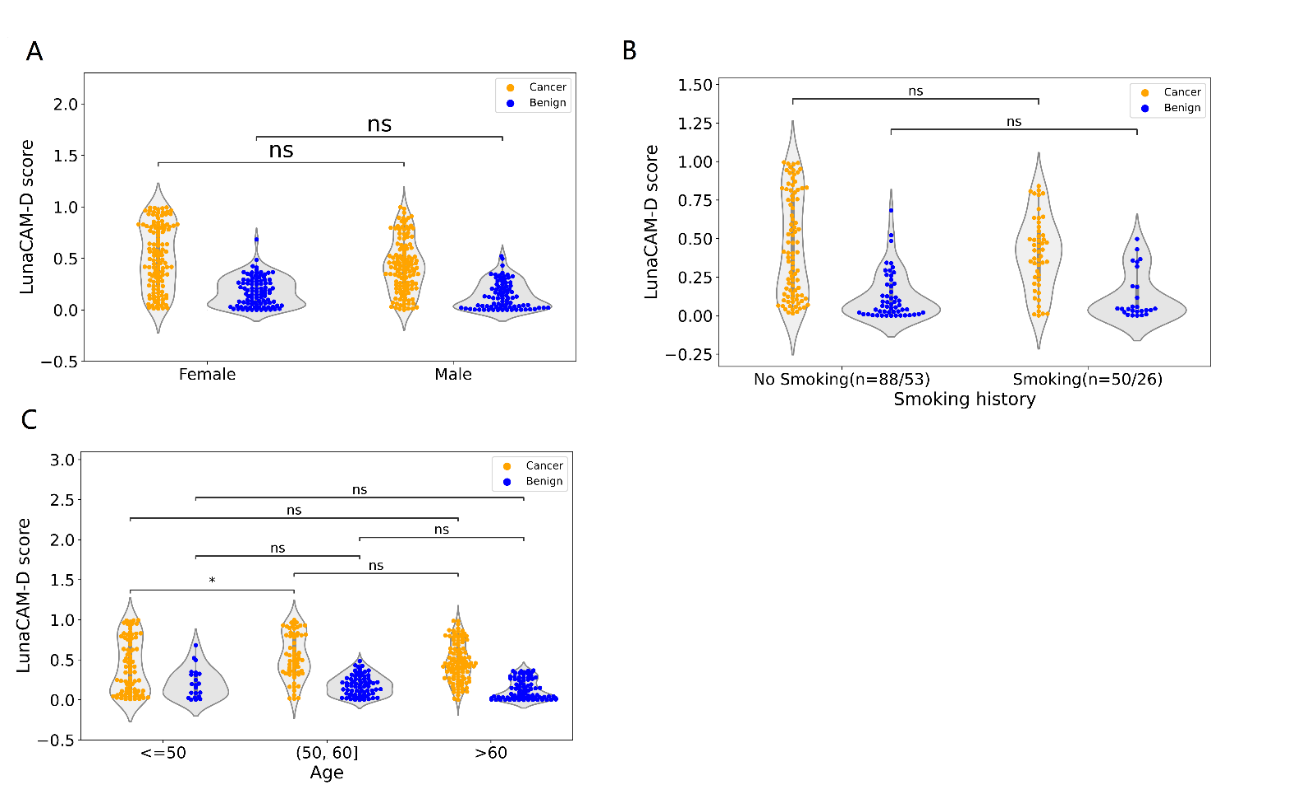


Supplementary Figure S4. Covariant analysis of LunaCAM-D scores against gender (A), smoking history (B), and age (C). Mann-Whitney u-test was used: **ns**: not significant (*p* > 0.05), *****: 0.01 < *p* <= 0.05; ******: 0.001 < *p* <= 0.01; *******: 0.0001 < *p* <= 0.001; ********: *p* < 0.0001.


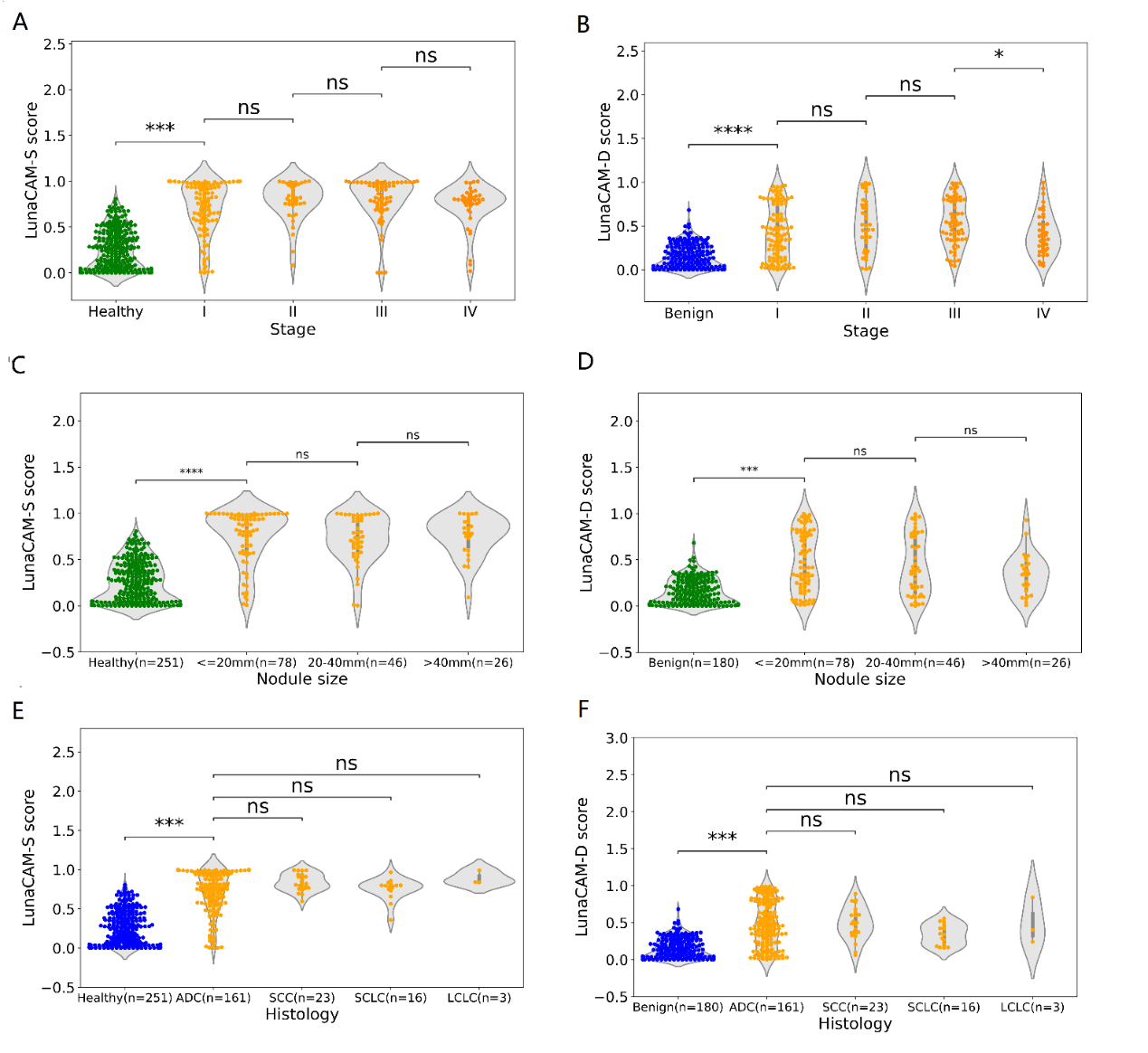


Supplementary Figure S5. Correlation of LunaCAM scores with clinical characteristics: A) LunaCAM-S scores in different stages; B) LunaCAM-D scores in different stages; C) LunaCAM-S scores in different nodule sizes; D) LunaCAM-D scores in different nodule sizes; E) LunaCAM-S scores in different lung cancer histology; F) LunaCAM-D scores in different lung cancer histology. Mann-Whitney u-test was used: **ns**: not significant (*p* > 0.05), *****: 0.01 < *p* <= 0.05; ******: 0.001 < *p* <= 0.01; *******: 0.0001 < *p* <= 0.001; ********: *p* < 0.0001.
